# Nanoparticle-Based Magnetic Resonance Imaging on Tumor-Associated Macrophages and Inflammation

**DOI:** 10.3389/fimmu.2017.00590

**Published:** 2017-05-22

**Authors:** Natalie J. Serkova

**Affiliations:** ^1^Department of Anesthesiology, Anschutz Medical Center, Aurora, CO, USA; ^2^Department of Radiology, Anschutz Medical Center, Aurora, CO, USA; ^3^Department of Radiation Oncology, Anschutz Medical Center, Aurora, CO, USA; ^4^Animal Imaging Shared Resources, University of Colorado Cancer Center, Anschutz Medical Center, Aurora, CO, USA

**Keywords:** magnetic resonance imaging, iron oxide nanoparticles, tumor-associated macrophages, inflammation, cancer

## Abstract

The inflammatory response, mediated by tissue-resident or newly recruited macrophages, is an underlying pathophysiological condition for many diseases, including diabetes, obesity, neurodegeneration, atherosclerosis, and cancer. Paradoxically, inflammation is a double-edged sword in oncology. Macrophages are, generally speaking, the major drivers of inflammatory insult. For many solid tumors, high density of cells expressing macrophage-associated markers have generally been found in association with a poor clinical outcome, characterized by inflamed microenvironment, a high level of dissemination and resistance to conventional chemotherapies. On another hand, radiation treatment also triggers an inflammatory response in tumors (often referred to as pseudoprogression), which can be associated with a positive treatment response. As such, non-invasive imaging of cancer inflammation and tumor-associated macrophages (TAMs) provides a revolutionary diagnostic tool and monitoring strategy for anti-inflammatory, immuno- and radiotherapies. Recently, quantitative T2-weighted magnetic resonance imaging (qT2wMRI), using injection of superparamagnetic iron oxide nanoparticles (SPIONs), has been reported for the assessment of TAMs non-invasively in animal models and in human trials. The SPIONs are magnetic resonance imaging (MRI) contrast agents that significantly decrease T2 MR relaxation times in inflamed tissues due to the macrophage-specific uptake and retention. It has been shown that macrophage-populated tumors and metastases will accumulate iron oxide nanoparticles and decrease T2-relaxation time that will result in a negative (dark) contrast in qT2wMRI. Non-invasive imaging of TAMs using SPION holds a great promise for staging the inflammatory microenvironment of primary and metastatic tumors as well monitoring the treatment response of cancer patients treated with radiation and immunotherapy.

## Introduction

The tumor microenvironment subsidizes to tumor progression, invasion, metabolic reprograming, and resistance to therapy. In the past decade, it has become increasingly clear that immune-competent cells, including macrophages, represent one of the main contributors to the aggressive tumor milieu (1, 2). Macrophages are phagocyting cells that penetrate into and reside in the affected tissue; they originate from circulating blood monocytes (3). Two distinct classes of macrophages have been described: classically activated M1 macrophages and alternatively activated M2 macrophages (4, 5). In most tumors, the inflamed microenvironment is driven by M2-type macrophages (6, 7).

Given the growing body of evidence of the tumor-associated macrophages (TAMs) playing a significant role in tumorigenesis, a non-invasive assessment of TAMs to detect the level of tumor inflammation becomes a critical and limiting factor. The gold standard for TAM assessment, as of now, is immunohistochemistry, histological examination, and, in rare case, flow cytometry on excised biopsies—all techniques can be applied *ex vivo* only (8). A biopsy comes at great costs to the patient, its invasiveness can have detrimental health consequences, and since it is very challenging, if not impossible, to perform sequential biopsies, these protocols are limited in the assessment of TAM infiltration over time. Unfortunately, the circulating levels of monocytes, which can be assessed by semi-invasive venipuncture (phlebotomy), do not bear any therapeutic values for correlating with the levels of TAMs. Molecular and cellular imaging is a fast growing area in translational and clinical research. Dozens of novel molecularly targeted imaging probes have been tested in animal models of cancer and some of them are successfully used in human imaging (9–11). Fortunately, macrophages are well known as “professional phagocytes” and are responsible for “cleaning” various exogenous microbes, toxins, and nanoparticles. Also, macrophages are responsible for endogenous and exogenous iron metabolism. Fortunate again, iron-based nanoparticles have been known as T2-weighted contrast for magnetic resonance imaging (MRI). The recent studies have shown that superparamagnetic iron oxide nanoparticles (SPIONs) have a potential for non-invasive T2-weighted MRI assessment on tissue residential macrophages, including TAMs (12). This approach has a high-translational potential, since several of the existing SPION agents are approved in Europe for MR imaging and commercially available for human use. Ferumoxytol is another ultrasmall SPION formulation (with an average particle size of 25–30 nm) that is approved by the US Food and Drug Administration as an iron supplement for intravenous treatment of iron deficiency in renal failure patients (13). Ferumoxytol has superb magnetic properties and has been safely used in animal and human trials as an off-label MRI contrast agent (12, 14–22).

## TAMs and Inflammation in Cancer

The relationship between chronic inflammation and cancer development was recognized well before the molecular origins of both diseases had been deciphered—Rudolph Virchow postulated an association between these two diseases in the 1860s (23). Inflammation is triggered by a cellular response, mediated mostly by neutrophils and macrophages, in response to pathophysiological stimuli. In general, macrophages are large white blood cells that ingest pathogens, microbes, and other invading substances. While neutrophils represent the first immune defense during the acute inflammation stage, the macrophages are predominant in chronic inflammation. All macrophages, including TAMs, are recruited through the local expression of chemoattractant stimuli such as macrophage chemoattractant protein 1 and colony-stimulating factor 1 (24–27). Overexpression of both these factors is correlated with poor prognosis in various tumors, including most aggressive breast cancer, pancreatic adenocarcinomas, lung cancer, and high-grade gliomas. In many solid tumor types, poor prognosis directly correlates with the abundance of TAMs. In breast cancer, for example, TAMs play a crucial role in epithelial/stromal cross talk, as shown for invasive ductal carcinoma and ductal carcinoma *in situ* (28). Macrophage depletion in animal models leads to the impaired lung tumor growth and the decreased metastatic spread to the lung from breast cancer (29–31). TAMs have been directly linked to matrix remodeling, angiogenesis, stimulation of tumor growth, and motility (27, 32, 33)—all these functions are also reported during wound healing; as such, tumors are often described as “wounds that never heal” (1, 7). Similar to Virchow, our contemporary scientists, Gonda et al. concluded that chronic inflammation results in a myriad of molecular event that produce a microenvironment that is favorable for the development of cancer (34). Agents that control the inflammatory cascade, such as ibuprofen and other non-steroidal anti-inflammatory drugs, are thought to reduce cancer risk or enhance other anticancer treatments.

In another scenario, TAMs can be recruited from circulating monocytes as a result of therapy-induced apoptosis resulting in tumor inflammation after, for example, radiation or chemotherapy. Radiation induces a genetic signature of chronic inflammation, which is enriched in genes regulating transendothelial migration, monocyte maturation, and leukocyte chemoattraction (35–37). In this case, surprisingly, the recruited macrophages can accelerate antitumoral effects of radiation treatment (38). As such, a non-invasive assessment of TAMs can serve a surrogate marker for a specific and early response to several anticancer therapies.

Recently, several phenotypes (or “states”) of macrophages/macrophage activation have been identified: two most extreme states are known as antitumor M1 and protumor M2 macrophages (39). M2-type TAMs promote tumor growth, angiogenesis, and metastases by promoting high-level expression of epidermal growth factor receptor and secretion of vascular endothelial growth factors. M1 phenotype, on the other hand, can directly or indirectly mediate tumor phagocytosis. Two classes have distinct molecular signatures—the antitumor M1 phenotype has relatively low IL-10 and high IL-12 expression, whereas protumor M2 macrophages express high IL-10 and low IL-12 levels. It has been hypothesized that the bad “protumor” M2 phenotype is responsible for intrinsically inflamed solid tumors promoting fast cell proliferation, angiogenesis, dissemination, and immunosuppression, while the good M1 macrophages mature in response to radiation and chemotherapy and, as such, can help other immunocompetent T-cells to recognized and fight cancer (40, 41). Nevertheless, clinically, most of the current IHC protocols are not capable to distinguish between two TAM phenotypes. Hence, it becomes increasingly imperative to non-invasively characterize patient’s tumor microenvironment for the presence of TAMs in order to stratify the patients to TAM-depleting and/or -directed therapies and to repeatedly monitor their treatment response.

## SPIONs and Iron Metabolism

The cytoplasm of a macrophage contains granules (also called packets) consisting of several enzymes and chemicals that are wrapped in a membrane; its membrane has an arsenal of highly effective scavenger receptors. They allow the macrophage to engulf a broad spectrum of invading microorganisms, pathogens, and nanoparticles as well as endogenous cell debris and apoptotic bodies. In fact, macrophages are known as “professional phagocytes” (42), and their phagocyting and pro-inflammatory abilities are directly linked to each other. There are some differences in terminology, which are related to the size of the digested material—some nanoscientists have introduced the term of “pinocytosis” for the uptake of soluble material or a nanoparticle, in contrast to the uptake of large material (“phagocytosis”) (43). But the fact remains undisputable—the macrophages take their responsibility of engulfing large and small nanoparticles very seriously; their macrophage scavenger receptors represent a very efficient system for recognizing a broad spectrum of surface modification and coating.

Fortunately for the MRI scientific community, iron-based nanoparticles represent even a higher level of attraction for the hard-working macrophages. Indeed, the macrophages are trained to maintain endogenous iron homeostasis while recycling and storing iron from senescent erythrocytes and other damaged cells (44, 45). They are very capable to store excessive levels of iron and, in response to systemic iron requirements, they can also release iron from their intracellular compartment into plasma. Therefore, after digesting (pinocyting) an iron-containing nanoparticle (SPION), a macrophage will dutifully retain iron as long as the circulating iron load remains within its physiological range. This macrophage-retained iron load can be easily detected by T2-weighted MRI.

## T2 Contrast in MRI

Modern oncologic imaging offers a variety of different modalities for the non-invasive detection and characterization of cancerous lesions (46–50). The common modalities include MRI, computed tomography, ultrasound, positron emission tomography (PET), single-photon emission computerized tomography, and optical imaging. Each modality has its advantages and disadvantages (46) and offers various non-invasive imaging endpoints related to tumor dimensions, tissue cellularity, angiogenesis, cancer metabolism, proliferation, and metastatic spread, just to mention a few. MRI is a non-invasive radiological technique with no ionizing radiation and high-spatial resolution, which is widely clinically used to detect, follow, and characterize solid tumors and metastases. MRI has complex physics and is based on physical properties of protons (mostly hydrogens) in a strong external magnetic field. Since water (H_2_O) is the main metabolite in all mammalian tissues, MRI detects small but distinct changes in spin frequencies of water hydrogens based on their surroundings when exposed to a high-magnetic field and radiofrequency excitation. The typical magnetic strength of human MRI scanner is 1.5 and 3 T, and for small animal imaging 4.7 and 7 T—however, the scanners tuned to even higher field, such as 9.4 T, 14 even 20 T, can be found in dedicated research facilities. One of the main strength of MRI is its ability to detect small changes (intrinsic contrast) within soft tissues and cell populations, which can be further enhanced by the use of intravenous contrast agents.

**Figure 1:**
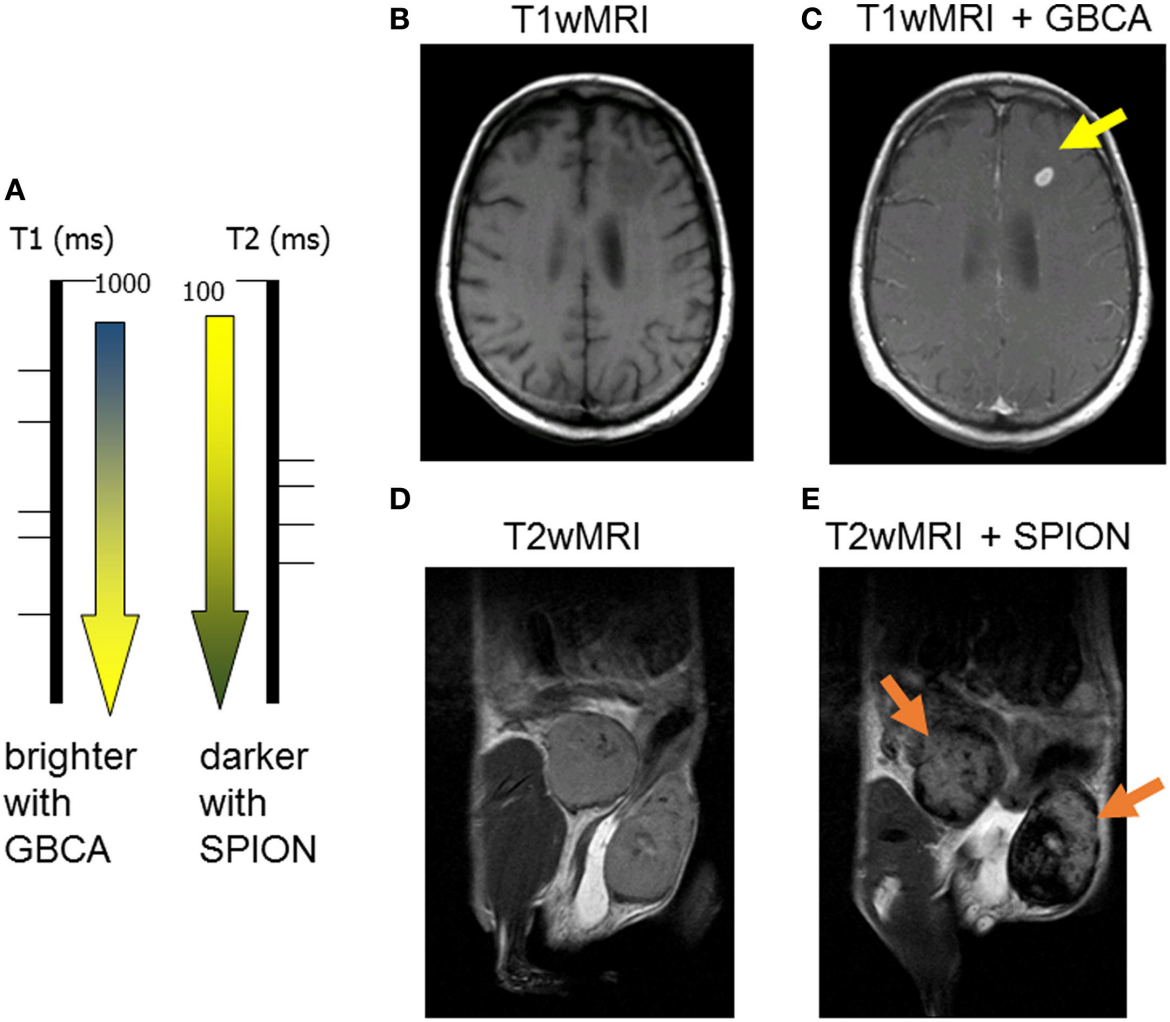
**Two major classes of magnetic resonance imaging (MRI) contrast agents: (A) paramagnetic gadolinium-based contrast agents (GBCA) are considered T1-positive contrast agents, by decreasing the spin–lattice T1 relaxation time, they produce bright T1 images; superparamagnetic iron oxide (SPION) is negative T2 contrast, iron oxide decreases the spin–spin T2-relaxation time producing darkening of T2-weighted images; (B) pre- and (C) post-GBCA T1-weighted MRI on a brain metastasis in a melanoma patient (15 min postinjection); (D) pre- and (E) post-SPION T2-weighted MRI on inflamed mouse mammary gland tumors (24 h postinjection)**.

It is important to understand the relationship between superparamagnetic nanoparticles and their effect on MR relaxation of the surrounding tissue water. Contrast agents can be principally divided into T1- and T2-relaxing contrast agents (Figure 1A). Paramagnetic contrast agents, such as gadolinium chelates also known as gadolinium-based contrast agents (GBCA), which are broadly used in the clinic, they predominantly shorten the spin–lattice T1 relaxation time (51). The shortening in T1 relaxation produces an increased signal intensity on a T1-weighted MRI images (Figures 1A–C). Clinically used GBCA (Magnevist, Omniscan, Mutihance, etc.) are intravascular contrast agents, not tailored to any specific cell type, and their specific accumulation in tumorous tissues is strictly based on liking vasculature of cancer. Figure 1C demonstrates an appearance of a small brain metastasis in GBCA-enhanced T1-weighted MRI (bright yellow arrow) in a melanoma patient.

On the other hand, all T2-shortening contrast agents consist of iron oxide nanoparticles, which are known as superparamagnetic (hence, the SPION abbreviation). By reducing the spin–spin T2-relaxation time of surrounding tissue water, the SPION (Feridex, Resovist, Ferumoxytol, etc.) produce darker contrast on T2-weighted MRI (Figures 1A,D,E) (52). Most importantly, unlike gadolinium, iron is a naturally occurring element in human bodies with low toxicity; and, the SPION are highly attractive to all phagocyting cells including macrophages (as well as Kupffer cells and the reticuloendothelial system, RES). The precise changes in T2-relaxation times (calculated from quantitative T2-weighted magnetic resonance imaging) can be used as a semi-quantitative assessment of TAM presence in a cancerous lesion. The Figure 1E shows a pronounced darkening in the inflamed mammary gland of a mouse by T2-weighted MRI (dark yellow arrows).

## Non-Invasive Imaging of TAMs Using SPIONs

Gadolinium-based contrast agents are, without any doubt, the major class of MRI contrast agents used in the clinic. Over the past 25 years, more than 100 million patients have undergone GBCA-enhanced T1-MRI. However, increased concerns about gadolinium deposition and toxicity of free gadolinium have impacted how GBCA are currently used. A severe side effect, known as nephrogenic systemic fibrosis, is associated with decreased renal clearance of GBCA in renally impaired patients (53), since acute toxicity of free gadolinium has been known for several decades. It can significantly limit the GBCA use for MRI in cancer patients with chemotherapy-induced low glomerulofiltration rate. Most recently, a very concerning study was published in *Radiology* on residual gadolinium deposition in the brain of patients after multiple GBCA injections for MRI (54). Alternative contrast agents to gadolinium chelates are being discussed, and the SPIONs are being increasingly used for various clinical scenarios in the recent years (20, 55).

Initially, all SPIONs were used for diagnostic liver imaging (mostly in hepatocellular carcinoma or liver metastases) and considered as safe MRI contrast (56). Their use was based on the high uptake of the SPION by the Kupffer cells: a drop of T2 signal was seen in normal hepatic parenchyma due to the Kupffer cell uptake, with no signal changes in liver lesions. There were also attempt to stage lymph node metastases due to SPION retention and phagocytosis in the RES and lymph nodes (57, 58). As all nanoparticles, SPIONs have enhanced permeability and retention (EPR) in solid tumors (52). However, the previous generation of SPION with larger particle sizes (around and above 50 nm) had been mostly captured by the RES, decreasing their half-life times and, as such, their penetration into tumors.

**Figure 2:**
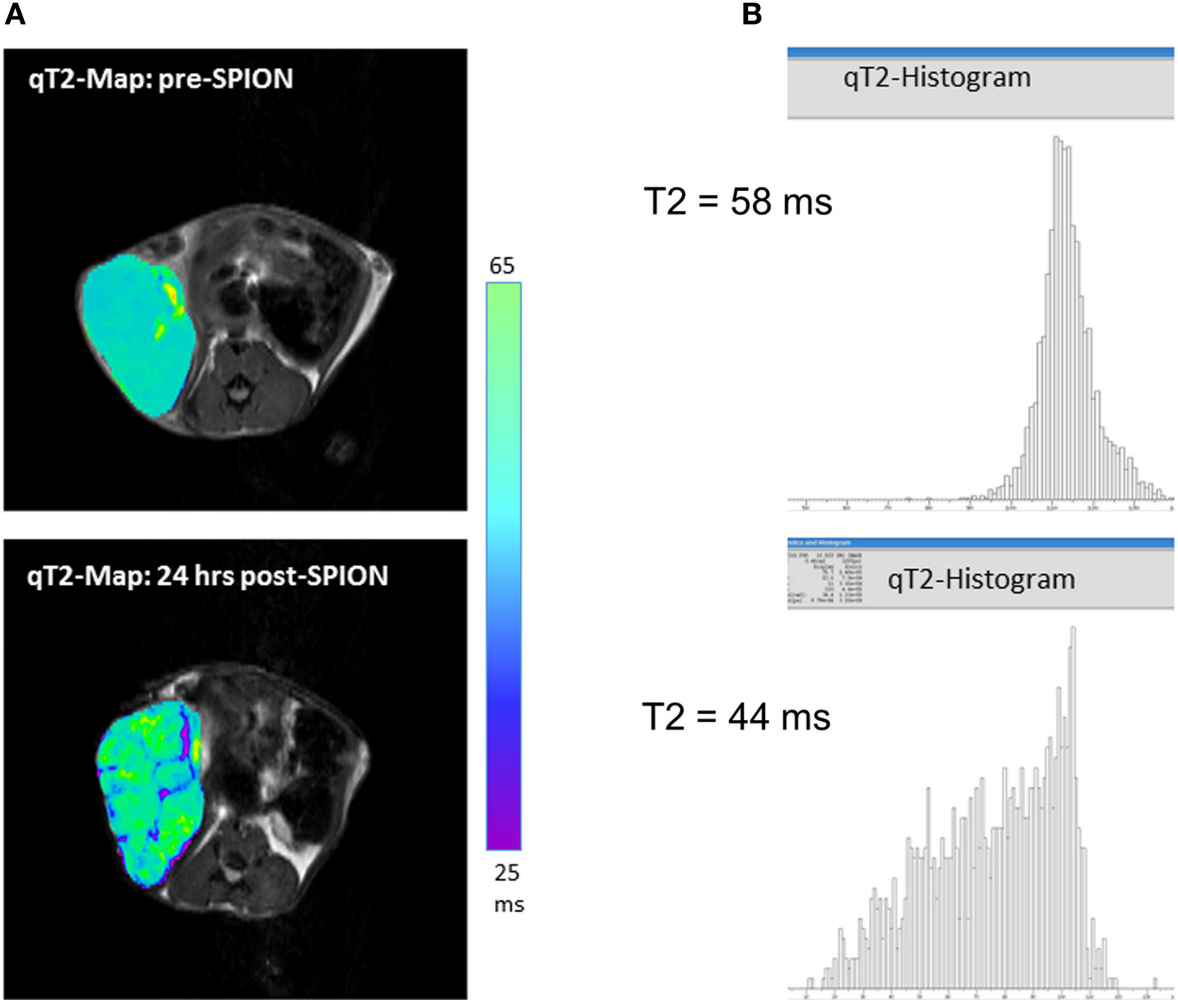
**(A)** T2-weighted magnetic resonance imaging (MRI) maps showing spatial distribution of nanoparticles (as dark signal intensities) in a high-grade glioma inflamed allograft in a mouse; **(B)** quantitative assessment of T2-weighted MRI presented as spatial T2 histograms with a T2 pre-contrast tumoral value of 58 ms; and 44 ms post-contrast. Pre-contrast images/histograms (top) and post-superparamagnetic iron oxide nanoparticle data (bottom) are 24 h apart.

Ferumoxytol is a colloid-based ultrasmall SPION, which consists of an iron oxide core with a size of ca. 6 nm and a carboxymethyldextran coat, resulting in a hydrodynamic diameter of 30 nm. Unlike larger SPIONs (e.g., Resovist), ferumoxytol has a prolonged circulating half-life time (>14 h in humans and 2 h in rodents), mostly because of it partially escaping phagocytosis by the RES (spleen, liver, and bone marrow). As such, ferumoxytol has a favorable EPR profile and a potential for higher tumoral biodistribution. It slowly leaks across highly permeable tumor vasculature into the tumor interstitial space; after that, ferumoxytol nanoparticles are attacked by the TAMs and slowly phagocyted—a process that takes hours. Our studies and those from others have shown that the pick of iron accumulation in a tumor (*T*_max_) lies between 16 and 24 h after intravenous injection of ferumoxytol (12, 15, 59). Figure 2A shows representative quantitative T2-MRI maps of a high-grade inflamed glioma allograft (a mouse flank model) before (top) and 24 h after ferumoxytol infusion (bottom). The T2 histograms on Figure 2B show a clear decrease in tumoral T2-rerelaxation times after 24 h of SPION injection (from 58 to 44 ms), with all iron being completely localized intracellularly in TAMs. The same effect can be seen in humans; a residual reduction in T2-relaxation times in inflamed cancerous lesions can sometimes be observed a week after ferumoxytol administration (15, 19, 60).

## Conclusion and Future Directions

Nanotechnology and nanomedicine have been increasingly utilized in translational and clinical practice in the past decade. This development has been supported by both federal and pharmaceutical funds, ever since, in 2004, the National Cancer Institute announced the Alliance for Nanotechnology in Cancer (61). The majority of nanoparticle research in cancer is focused on the targeted drug delivery of chemotherapeutic drugs, mostly for increasing tumor accumulation and decreasing systemic toxicity (62). Recently, an exciting area of nanomedicine has evolved, known as theranostics, based on the idea that the same drug carriers can be design as potent imaging agents (63–65). SPIONs are increasingly used for T2wMRI in oncology, including fast-evolving macrophage imaging. Macrophage-driven uptake of iron allows for the non-invasive assessment of the tissue inflammation status in cancer, diabetes, and ischemia/reperfusion injury. In the future, the same SPIONs can be loaded with an anti-inflammatory or chemotherapeutic agent to selectively deliver a therapy to the inflamed lesion.

An alternative to the use of SPION agents is the use of perfluocarbons that can be visualized for fluorine (^19^F) magnetic resonance spectroscopy (MRS) for cell tracking of inflammatory cells (66). Some limitations to the ^19^F-MRS application include low sensitivity to the target (usually, in the millimolars range). Another alternative might arise from the use of hyperpolarized ^13^C-arginine by ^13^C-MRS (67), since upregulated expression of arginase has been found in M2-like macrophages. However, the hyperpolarized ^13^C-MRS approach is technically and clinically challenging and available only at the very limited number of academic hospitals. For PET, the uptake of radioactive glucose analog (18F-fluoro-deoxyglucose) by inflamed tissue is well known, but unfortunately, is rather non-specific since the tumor cells also have elevated glucose uptake (68, 69). Most recent studies try to use a specific ^18^F-tracer for the translocator protein to image activated microglial cells and, possibly, TAMs in inflamed gliomas (70). But, as of today, the SPION-based T2-MRI approach appears to be clinically the most feasible path to image TAMs and to follow the response to anti-inflammatory treatment non-invasively. In the future, the combined PET/MRI might be the best available option for human imaging, since the first multimodality scanners have recently became available (71). Ideally, the future imaging studies should be designed to non-invasively discriminate the protumor M2 versus antitumor M1 macrophages, since this phenotyping might play a crucial role in assessing tumor response to novel checkpoint inhibitors and other immunotherapies (72). A non-invasive TAM imaging will enable to characterize the inflamed tumor microenvironment, selectively deliver novel anti-inflammatory and anticancer drugs, and monitor their efficacy non-invasively and in the real time, providing new horizons for oncological imaging well above the limitations of conventional “volumetric” criteria.

## Author Notes

The original data provided in Figures 1 and 2 were acquired in NS group.

## Author Contributions

NS was solely responsible for literature search and composing this manuscript.

## Conflict of Interest Statement

The author declares that the research was conducted in the absence of any commercial or financial relationships that could be construed as a potential conflict of interest. The reviewer, LD, and handling editor declared their shared affiliation, and the handling editor states that the process nevertheless met the standards of a fair and objective review.
